# Hidden toll of violent deaths during pregnancy and the postpartum period: a nationwide analysis of Mexican death records

**DOI:** 10.1136/bmjph-2025-004871

**Published:** 2026-05-13

**Authors:** Ursula Gazeley, Maria Gargiulo, Hallie Eilerts-Spinelli, Anushé Hassan, Itzel Díaz-Juárez, Alexis Palfreyman

**Affiliations:** 1 Nuffield Department of Population Health, University of Oxford, Oxford, UK; 2 Nuffield College, University of Oxford, Oxford, UK; 3 Department of Population Health, London School of Hygiene & Tropical Medicine, London, UK; 4 International Health, Johns Hopkins University, Baltimore, Maryland, USA; 5 El Colegio de México, Mexico City, Mexico; 6 Centre for Impact on Violence and Health, Colombo, Sri Lanka

**Keywords:** Violence, Mental Health, Female, statistics and numerical data

## Abstract

**Introduction:**

Violent deaths during pregnancy and the postpartum period remain an unmet global public health priority. These include deaths from self-inflicted (ie, suicide) and externally perpetrated violence (including deaths due to assault, legal intervention and operations of war or of undetermined intent). Mexico—where both the levels of violence and data completeness are high—offers a unique opportunity to assess how current international maternal surveillance frameworks obscure the burden of lethal violence during this period.

**Methods:**

We analysed data on all deaths of pregnant and postpartum women, up to one year following the end of pregnancy, regardless of birth outcome, from the implementation of International Classification of Diseases, 10th Revision (ICD-10) onwards (1998–2024) in Mexico. Deaths from violence were compared with maternal and late maternal causes, categorised by ICD-Maternal Mortality grouping. Modalities of violence were compared by timing of death (pregnant or postpartum).

**Results:**

Between 1998 and 2024, official statistics recorded 36 662 pregnancy-related deaths of women aged 10–54 in Mexico, of which 1855 were due to violence: 1287 from external violence (assault: n=1133, undetermined intent: n=154) and 568 from self-inflicted violence. Violent deaths increased since 2010. Across the study period, they surpassed or were comparable to leading maternal obstetric causes: exceeding pregnancy-related infection (n=929) and approaching those from abortive outcome (n=2101). The leading modality of external violence was firearm-related death (n=650) followed by hanging/strangulation (n=206), whereas hanging/strangulation (n=423) was most common among self-inflicted deaths followed by self-poisoning (n=104).

**Conclusions:**

Violence during pregnancy and the postpartum period remains an overlooked public health concern, insufficiently prioritised within maternal health and violence prevention agendas. International definitions and existing surveillance systems fail to capture the burden of these violent deaths: suicides need to be better reported within maternal death statistics, while a new indicator is needed to explicitly monitor homicides during pregnancy and the postpartum period. Robust vital statistics data from Mexico underscore the urgent need to integrate violence prevention into maternal health frameworks, strengthen clinical screening and address the structural determinants of suicide and homicide during pregnancy and the postpartum period.

**WHAT IS ALREADY KNOWN ON THIS TOPIC**

**WHAT THIS STUDY ADDS**

- This study quantifies violent deaths relative to obstetric causes in Mexico, highlighting their exclusion from maternal mortality statistics: suicide by default, homicide by design.
- Mexico’s high burden of violence and robust vital statistics system make it a useful case study for examining how International Classification of Diseases definitions shape global maternal mortality surveillance.

**HOW THIS STUDY MIGHT AFFECT RESEARCH, PRACTICE OR POLICY**

- Violent deaths during pregnancy and the postpartum period are under-recognised from both the maternal health and violence agendas; prevention requires both clinical action through antenatal and extended postpartum care and structural action on gender-based violence, poverty and reproductive autonomy.
- Suicide reporting within maternal mortality statistics needs strengthening, and a dedicated global indicator for homicides of pregnant and postpartum women is needed to ensure these deaths are counted.

## Background

 Violence—whether self-inflicted or externally perpetrated—remains an overlooked cause of death during pregnancy and the postpartum period. Global maternal mortality frameworks, based on the International Classification of Diseases (ICD), define a maternal death as occurring during pregnancy or within 42 days post partum, from any cause related to or aggravated by pregnancy, excluding accidental or incidental causes.^1^ This definition includes suicides but excludes deaths due to external violence, such as homicides. Only deaths meeting the ICD definition are included in the maternal mortality ratio (MMR), the indicator used to monitor progress towards the Sustainable Development Goal (SDG) 3.1, which sets a global target of fewer than 70 maternal deaths per 100 000 live births by 2030.^1^ This distinction between suicide and external violence has far-reaching implications for how health systems and international agencies recognise and respond to violence during and after pregnancy.

Under the ICD-Maternal Mortality (ICD-MM) grouping system, maternal suicides are classified as ‘other direct maternal causes’, acknowledging possible links between pregnancy, mental disorders and suicide risk.^2^ Suicide is a leading cause of maternal death both in countries with robust surveillance systems, such as the UK, and lower resource contexts using periodic reproductive age mortality studies.^3 4^ Yet, across settings, maternal suicides are consistently under-recorded due to stigma.^5 6^ Additionally, even in countries with functioning civil registration and vital statistics (CRVS) systems, incomplete reporting of pregnancy status contributes to the misclassification of suicides and other deaths as non-maternal.^6 7^

Homicides of pregnant and postpartum women, by contrast, are considered incidental to pregnancy and are therefore explicitly excluded from maternal mortality statistics. While violent deaths occurring within 42 days post partum may be captured in countries’ pregnancy-related mortality statistics (all-cause mortality during pregnancy or within 42 days post partum), pregnancy-related deaths including homicides beyond this window are not captured under any existing indicator,^8 9^ further obscuring their burden, especially for marginalised groups.^10 11^ These definitional boundaries mirror fragmentation between global maternal health, mental health and gender-based violence measurement systems, research, practice and advocacy.^5^

The exclusion of violent deaths from maternal mortality persists despite evidence that pregnancy and the post partum may be periods of heightened vulnerability to violence.^12^ An unwanted pregnancy,^9^ honour killings due to family disapproval,^13^ financial stress and an infant’s illness or sex might trigger the initiation or escalation of abuse,^12^ suggesting violence can be socially ‘aggravated’ by pregnancy. The shared structural and social determinants of community-level conflict with intimate partner^14^ and obstetric^15^ violence likewise contribute to negative maternal outcomes.^16^ Suicide may also be a last resort for women experiencing domestic or interpersonal violence or an unwanted pregnancy.^9 17^ Recognising these intersections highlights the importance of analysing these forms of violence and maternal health together.^9 17^

Mexico offers a unique case to examine the implications of this measurement decision due to its current context of persistently high levels of interpersonal, gender-based and community-level violence. Since the late 2000s, homicides have contributed to stagnating life expectancy.^18^ Feminicide specifically has become an increasingly important determinant of female life expectancy,^19^ although existing statistics likely undercount the true burden of feminicide in Mexico^20^ and globally due to data quality challenges.^21^ Additionally, since 2023, pregnancy is considered an aggravating factor for feminicide in Article 325 of Mexico’s Federal Criminal Code (D.O.F. 25-04-2023). At the same time, Mexico’s CRVS system—among the most complete in Latin America^22^—features high-quality death registration, consistent use of ICD-10 and a pregnancy checkbox on death certificates to facilitate the identification of deaths occurring during pregnancy and the postpartum period, regardless of cause.^23^ As an upper-middle-income country,^24^ these features make Mexico one of the few low- and middle-income countries with sufficient data to analyse deaths due to both self-inflicted and external violence in relation to pregnancy.

Current maternal mortality definitions capture only part of the continuum of violence experienced during and after pregnancy: suicides are inconsistently recorded and homicides are systematically excluded. Using data from Mexico—where both the burden of violence and data completeness are high—we aimed to examine how the exclusion of violent deaths from maternal mortality frameworks obscures the true burden of lethal violence during pregnancy and the postpartum period.

## Methods

All analyses were conducted using R and all code is available online (https://doi.org/10.17605/OSF.IO/DPFGV).

### Data sources

We used de-identified birth and death certificate microdata from 1998 to 2024, published by the *Instituto Nacional de Estadística y Geografía* (INEGI), to calculate the number of live births^25^ and the number of deaths^26^ among pregnant and postpartum women during the period of study.

### Coding pregnancy status

A pregnancy checkbox has been included on death certificates in Mexico throughout the period of study. From 1998 to 2003, the pregnancy checkbox question was to be considered for any female death, and it included two categories indicating whether the decedent was pregnant or postpartum: (1) ‘within 42 days prior to their death’ or (2) ‘within 11 months prior to their death’. From 2004 onwards, the pregnancy checkbox was further expanded to indicate whether the death occurred: ‘during pregnancy’, ‘during labour’, ‘during the postpartum period’, ‘between 43 days and 11 months after the birth or an abortion’, or that the decedent was ‘not pregnant in the 11 months prior to the death’. The death certification form used between 2004 and 2011 specified that the pregnancy checkbox question should be answered in the case of the death of any woman of ‘fertile age’. Starting in 2012, the question specified that it should be answered in the case of the death of any woman between 10 and 54 years of age. Due to these changes in the death certificate form, we grouped information about pregnancy status at time of death into three categories: (1) death during pregnancy, the intrapartum period or within 42 days post partum, (2) death between 43 days and up to 1 year post partum and (3) not pregnant within the last year.

Consistent with national practices in Mexico, where the pregnancy checkbox is applied to women aged 10–54 years in vital statistics published by INEGI, we included this full age range. This extends the conventional definition of reproductive age (15–49 years) used by the WHO.^27^ Given evidence of elevated maternal mortality risk at both very young and older ages,^28^,^30^ this approach may reduce under-ascertainment of pregnancy-related deaths at the youngest and oldest ages.

### Cause of death classification

Causes of death were grouped according to ICD-MM^2^ categories for maternal causes, as follows: (1) pregnancy with abortive outcome, (2) hypertensive disorders of pregnancy, (3) obstetric haemorrhage, (4) pregnancy-related infection, (5) other obstetric complications, (6) unanticipated complications of management, (7) non-obstetric complications and (8) unknown or undetermined causes. Deaths assigned only to contributory conditions (such as obstructed labour) without an underlying cause were categorised separately from ICD-MM categories but were included in all analyses as maternal deaths. Deaths from late maternal causes and those coded to sequelae of obstetric causes (despite occurring within 1 year post partum) were grouped together. Violent deaths were classified separately from ICD-MM categories as either self-inflicted (deaths due to intentional self-harm) or externally perpetrated (deaths due to assault, legal intervention and operations of war, or of undetermined intent—the latter assumed to be predominantly externally inflicted).^31^ The list of ICD-10 codes for each category can be found in online supplemental table 1 (pp2–3). INEGI death certificate data includes additional fields (eg, ‘pregnancy-related’ and ‘maternal causes’ indicators) used in national statistics. However, because these categories do not fully align with ICD-MM, we used only ICD-MM for the classification of maternal deaths.

### Statistical analysis

We used descriptive analyses to examine the contribution of violent deaths over time relative to ICD-MM categories and identified the most common modalities of self-inflicted and externally perpetrated violence, by timing of death. We calculated MMRs using all maternal deaths in the ICD-MM categories and contributory conditions. We then recalculated the ratios, including deaths due to self-inflicted violence, deaths due to externally perpetrated violence and all violent deaths combined, to assess the magnitude of the impact of their exclusion. We analysed the spatial variation and correlations between external violence and suicide at the state level to identify high-burden states. Finally, we compared the burden and age distribution of external and self-inflicted violent deaths among pregnant and postpartum women with those among all women of reproductive age (10–54 years).

### Patient and public involvement

Patients or the public were not involved in the design, conduct, reporting or dissemination plans of our research.

## Results

### Pregnancy reporting

In total, from 1998 to 2024, there were over 1.3 million deaths among women of reproductive age in Mexico. Of these, there were 36 662 pregnancy-related deaths that occurred during pregnancy and up to 1 year post partum. However, approximately 28% (n=364 322) of all reproductive age deaths had missing information on the pregnancy checkbox and were excluded from the analyses. Most missing data occurred from 2015 onwards, which may indicate a change in coding practices (eg, conflation of the ‘not pregnant’ or ‘not applicable’ codes with the ‘not specified’ code) (see online supplemental figure 1, p4). Few of these reproductive age deaths (n=67) were coded to obstetric causes, but approximately 7% (n=27 005) of these were coded to ICD-10 causes indicating deaths due to self-inflicted or externally perpetrated violence. Sex was missing for approximately 0.06% (n=10 300) of all death certificates registered between 1998 and 2024, and age was missing for approximately 0.5% (n=87 058). The mean age of death for women who died during pregnancy or in the postpartum period was 29.2 years (SD 8.5 years). Online supplemental figure 2 (p5) shows the full age distribution.

### Violence

Of all pregnancy-related deaths, 1855 were due to violence: 69% (n=1287) were recorded as externally perpetrated (deaths due to assault (n=1133), legal intervention and operations of war (n=0) and events of undetermined intent (n=154)); 31% (n=568) were recorded as self-inflicted (deaths due to intentional self-harm). The number of deaths from violence increased in most years from 2011 onwards, except in 2020 and 2021 (see online supplemental figure 3 and table 2 on p6 and p7, respectively).

**Figure 1:**
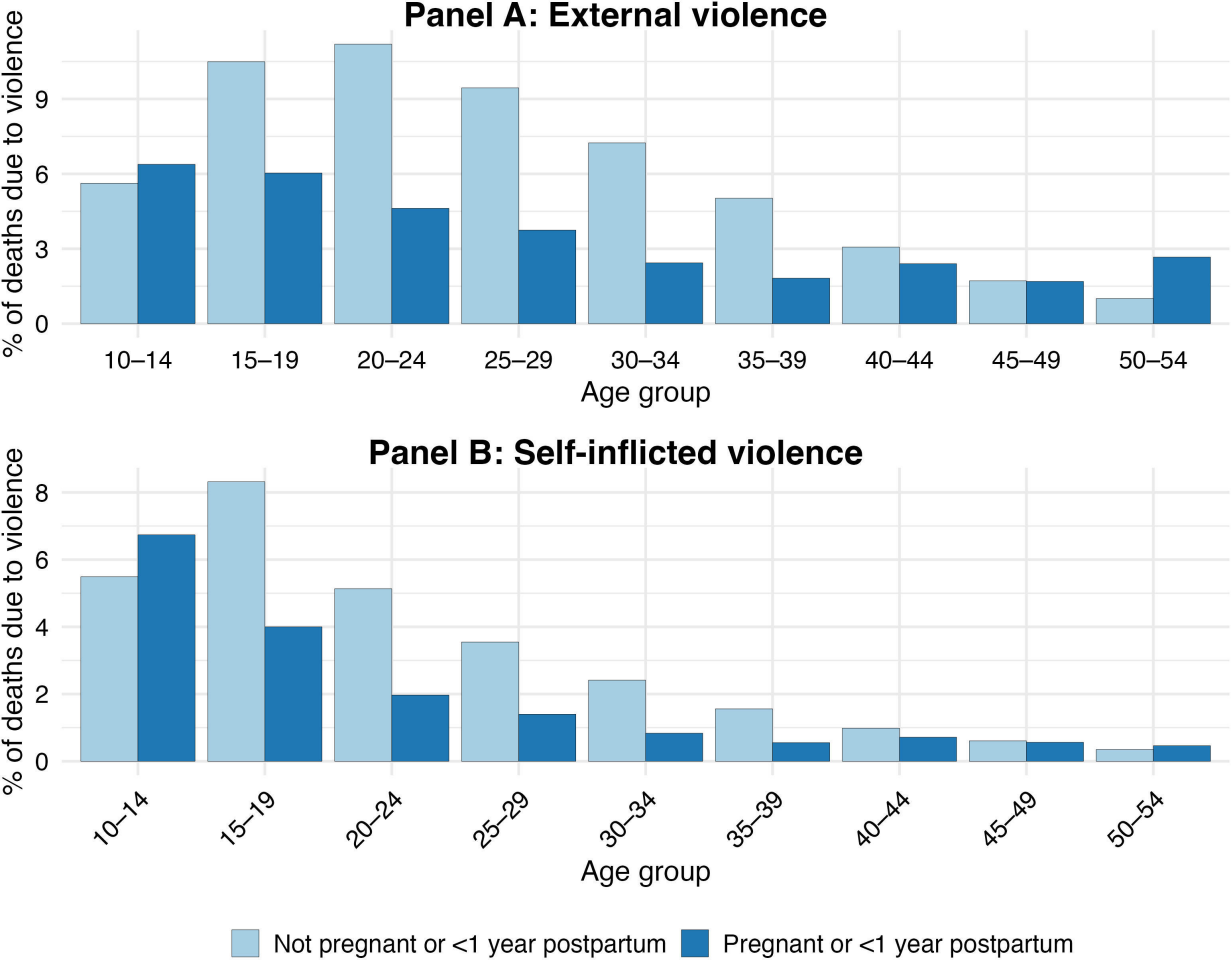
Comparison of self- and externally inflicted violence by age during pregnancy and post partum to non-pregnant and postpartum women of reproductive age (10–54 years) (1998–2024).

We compared the proportion of deaths due to violence (external and self-inflicted) among women reported as pregnant or within 1 year post partum with that among women of reproductive age (10–54 years) who were not reported as pregnant or within 1 year post partum on the death certificate. As shown in figure 1, the proportion of violent deaths was higher for pregnant and postpartum women at both ends of the age distribution (10–14, 50–54). For all other ages, the proportion of deaths due to violence was higher among women of reproductive age not reported as pregnant or postpartum on the death certificate, though this difference narrowed markedly from age 40 with convergence by age 45–49. See online supplemental table 3 (p8) for a tabular breakdown of this information.

### Relative burden of violence compared with maternal causes

Between 1998 and 2024, there were 25 919 maternal deaths and 1643 late maternal deaths (including 4 deaths occurring within 1 year post partum but coded to sequelae of obstetric causes). Late maternal deaths increased over time (see online supplemental figure 3, p6).

**Figure 2:**
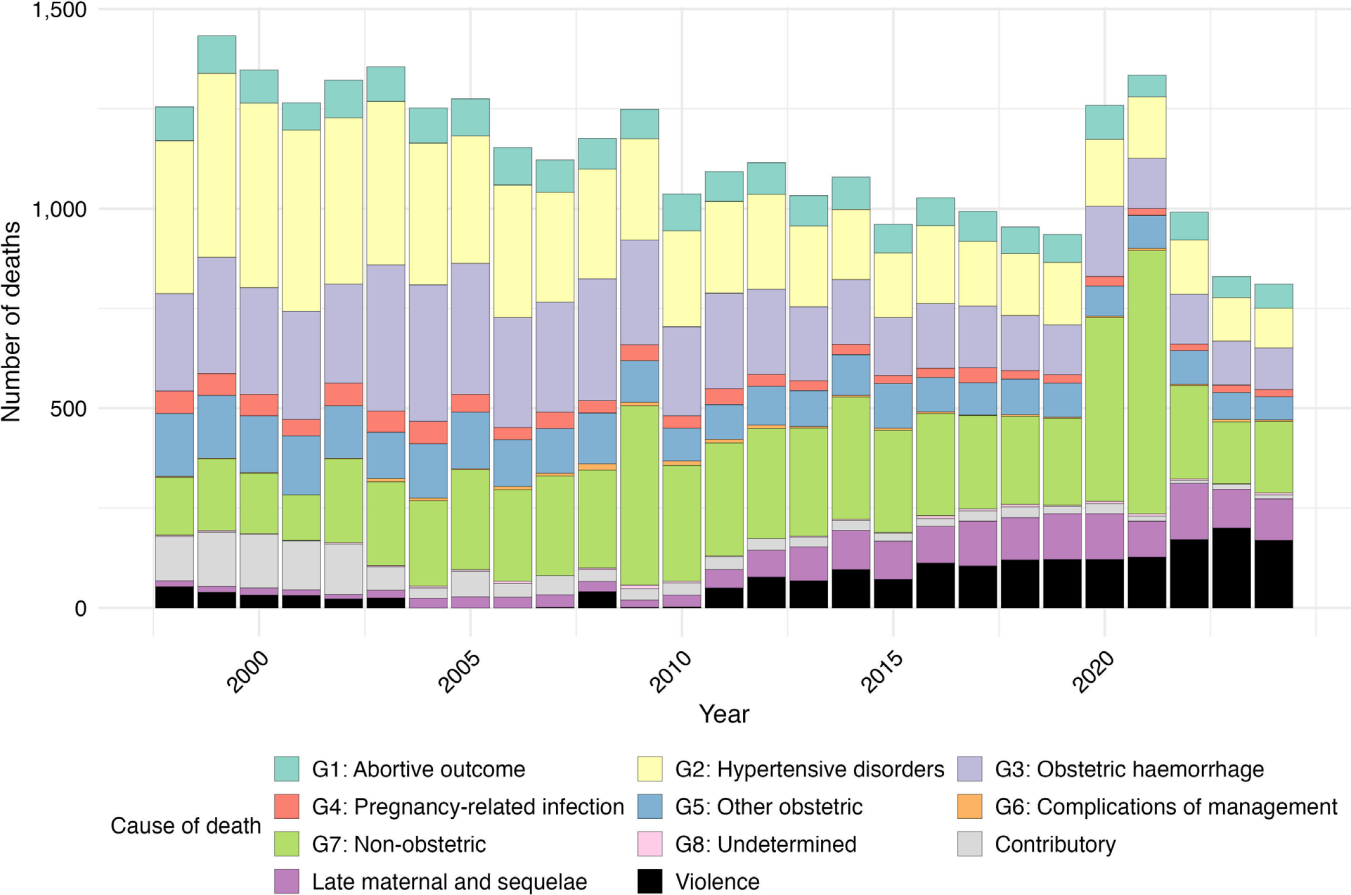
Deaths by International Classification of Diseases-Maternal Mortality group, late maternal causes, contributory causes and violence over time (1998–2024).

Figure 2 shows the burden of deaths from violence relative to maternal causes (ICD-MM groups) from 1998 to 2024. Violent causes (n=1855) exceeded those from several obstetric categories—group 4 (pregnancy-related infection; n=929), group 6 (complications of management; n=142) and group 8 (undetermined causes; n=101)—and approached those from group 1 (abortive outcome; n=2101). See online supplemental table 4 (pp9–10) for a tabular breakdown of this information.

Figure 2 also shows that there was an increase in the number of deaths during pregnancy and up to 1 year post partum from non-obstetric causes in 2009, 2020 and 2021. This group was predominantly composed of deaths coded to *O98: Maternal infectious and parasitic diseases classifiable elsewhere but complicating pregnancy, childbirth and the puerperium* (mostly *O98.5: other viral diseases complicating pregnancy*) and may reflect influenza A (H1N1) or COVID-19 deaths.

Between 1998 and 2024, there were 1239 deaths coded to ‘contributory conditions’, including obstructed labour, that are not underlying causes of death as specified by ICD-MM guidelines. The number of deaths coded to contributory conditions decreased over time, falling dramatically in the early 2000s (see online supplemental figure 4, p11).

### Modalities of violence

**Figure 3:**
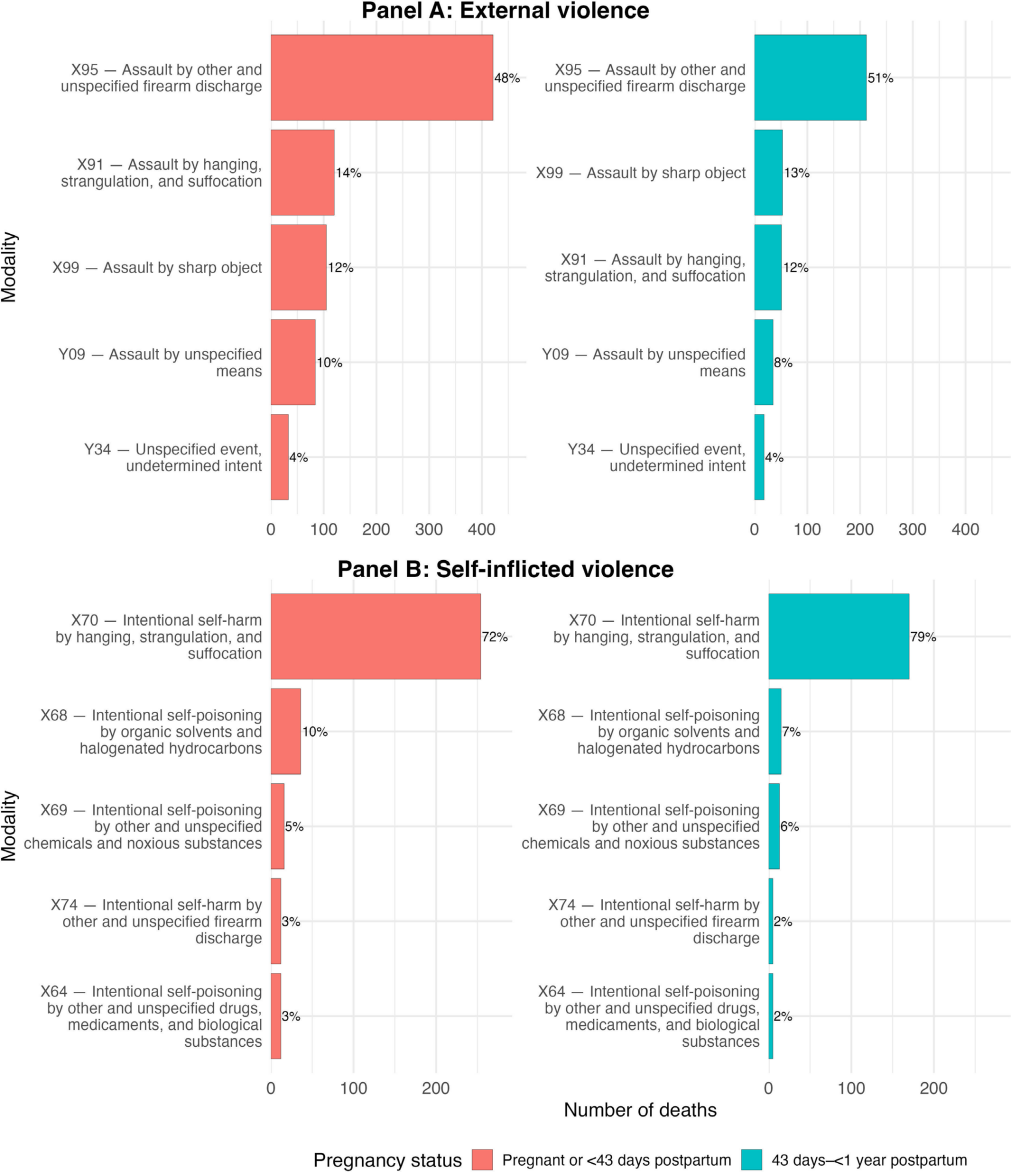
Modalities of violent deaths by pregnancy status at the time of death (1998–2024).

The most common modality of external violence was firearm killings (n=650, 51% of deaths due to external violence), followed by hanging and strangulation (n=206, 16%). The most common modality of self-inflicted violence was hanging and strangulation (n=423, 74% of deaths due to self-inflicted violence), followed by self-poisoning (n=104, 18%). As shown in figure 3, the modalities of violent deaths were broadly the same across the two groups of women: those who died during pregnancy and up to 42 days post partum, and those who died in the extended postpartum period from day 43 to 1 year. This finding was consistent if timing of death was further disaggregated to: during pregnancy (including intrapartum deaths that likely reflect misclassification of the time of death, though external or self-inflicted violence during labour cannot be excluded), early post partum up to 42 days and late post partum (see online supplemental figures 5 and 6 on p12 and p13, respectively).

There was little change in the modalities of external and self-inflicted violence over time, except for a slight shift towards the use of firearms for externally perpetrated deaths, and a shift towards hanging and away from poisoning for self-inflicted deaths (see online supplemental figure 7, p14).

Finally, we compared the modalities of violent deaths for women who were pregnant and postpartum to other women of reproductive age who were not reported as being pregnant or recently pregnant at the time of death. Overall, patterns of violence were broadly consistent, except that pregnant or postpartum women had a higher proportion of firearm-related external deaths and a higher proportion of hanging, strangulation and suffocation among self-inflicted violent deaths compared with other women of reproductive age (see online supplemental figure 8, p15).

### Absolute and relative changes in the MMR

**Figure 4:**
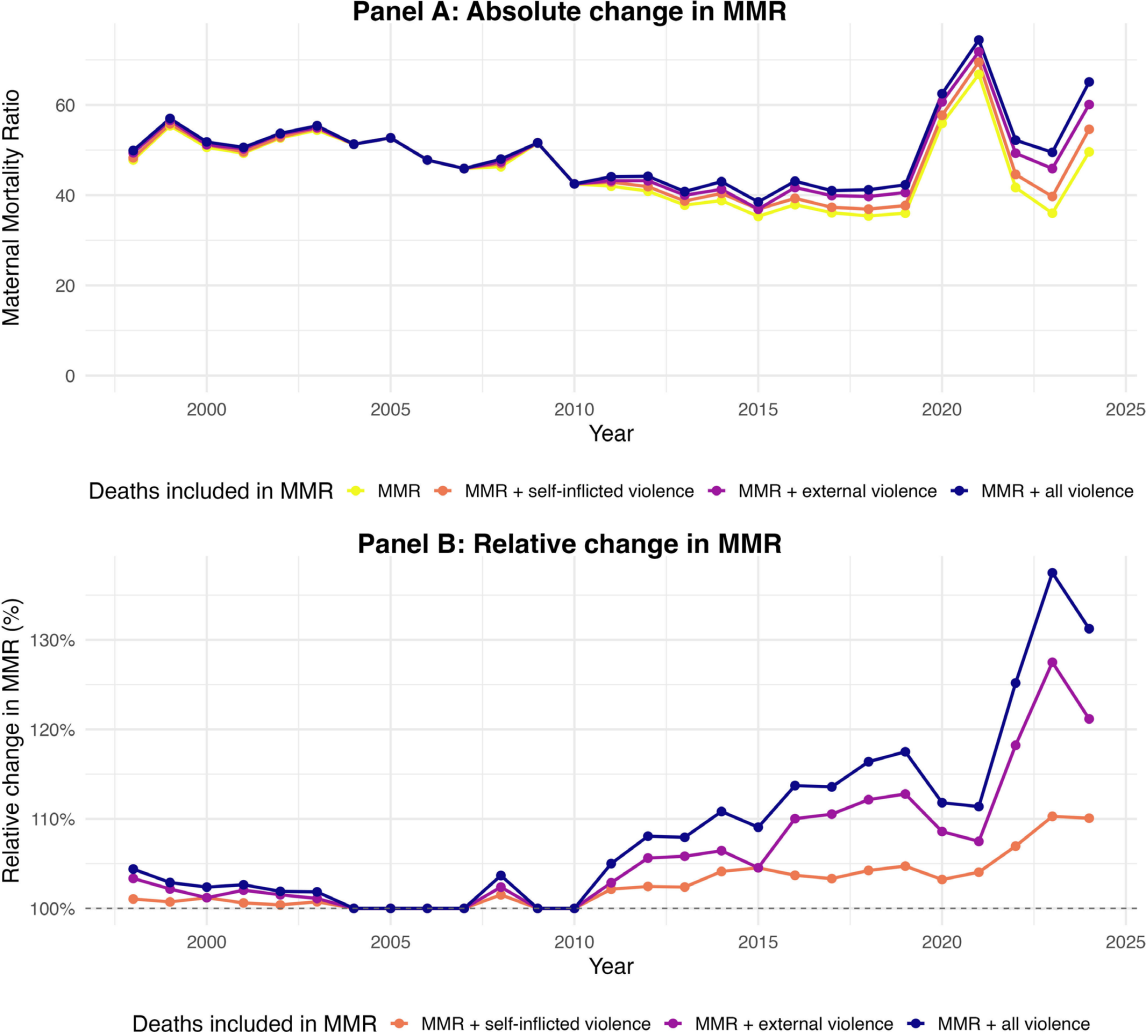
Absolute and relative changes in the MMR with inclusion of violent deaths during pregnancy and post partum (1998–2024). MMR, maternal mortality ratio.

Figure 4 shows the absolute (panel A) and relative (panel B) change in the MMR if deaths from self-inflicted violence, external violence and both were included. In 2024, the exclusion of suicide deaths from the maternal causes (counter to ICD-MM guidelines) means we would underestimate the MMR in Mexico by 5 per 100 000 live births, or roughly 10%. The absolute and relative change in the MMR incurred if deaths from external violence were included in the MMR is more substantial. The MMR in 2024 would increase from 50 per 100 000 live births to 60 per 100 000 if externally perpetrated violent deaths were included, corresponding to a 21% relative increase. If deaths due to both self-inflicted and external violence were included in the MMR, the MMR would increase to 65 per 100 000, corresponding to a relative increase of approximately 31%.

**Figure 5:**
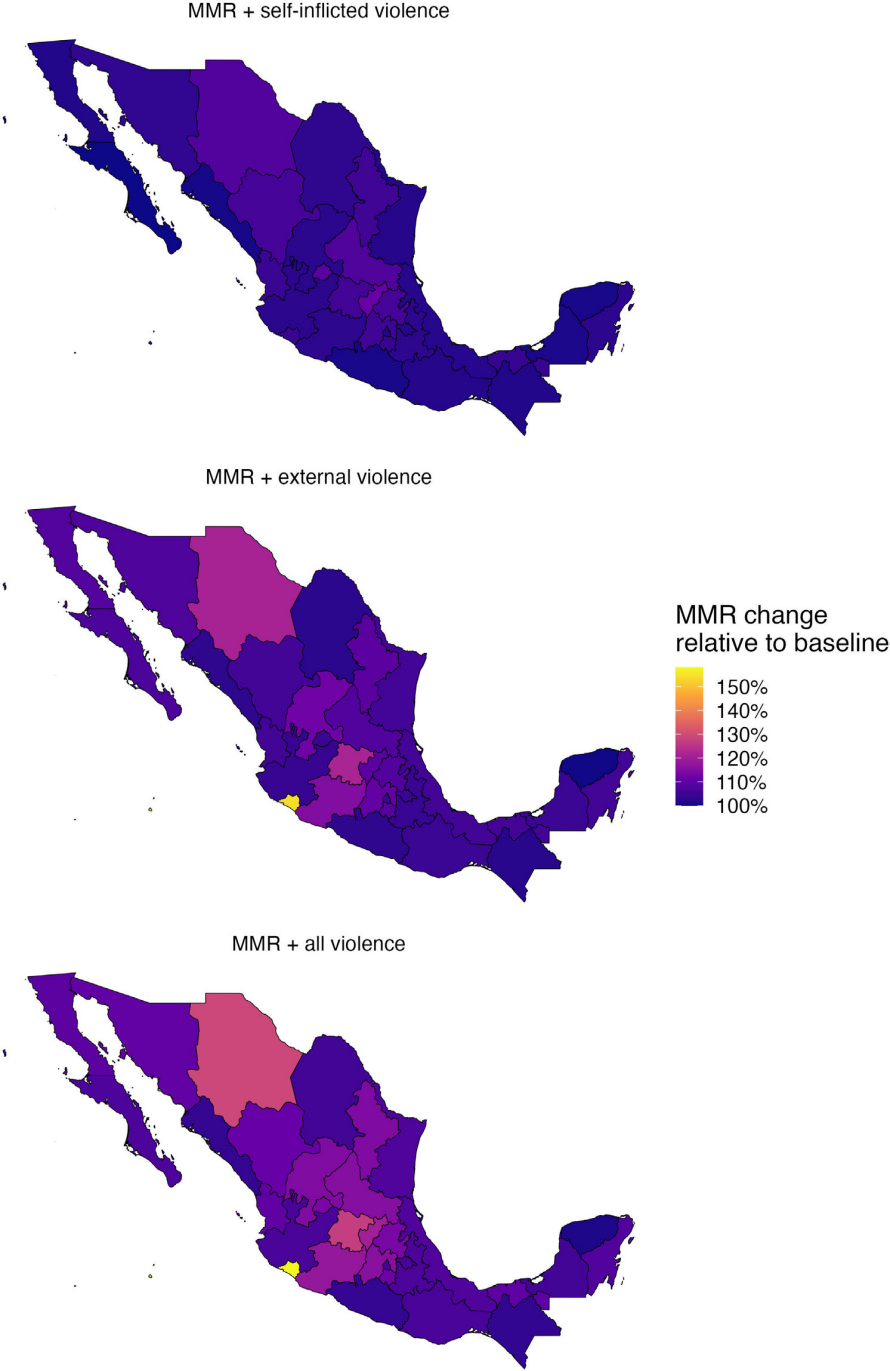
Spatial variability in the burden of self-inflicted and external violence by state (1998–2024). MMR, maternal mortality ratio.

Figure 5 presents the spatial distribution of violent deaths in Mexico for 1998–2024. There was much greater variability between states’ burden of homicide than suicide. The relative increase in the MMR if all violent deaths were included is greatest in Colima—a small, unpopulous state with small numbers of maternal and violent deaths—followed by Chihuahua and Guanajuato. For the strength of the spatial correlation between the burden of self-inflicted and external violent deaths, see online supplemental figures 9 and 10 (pp15–16). There was substantial variation in correlation between self-inflicted and external violent deaths over space and time (online supplemental figure 9, p16). Using all data from 1998 to 2024, there was a stronger correlation between deaths due to self-inflicted and external violence among women who were not pregnant or postpartum in the year prior to their death compared with those who were, especially in central states (online supplemental figure 10, p17).

## Discussion

The definition of maternal mortality determines which women’s deaths are visible to health systems. Decisions about the inclusion or exclusion of specific causes of death shape surveillance, accountability and the allocation of resources within maternal health policy.^9 32^

Using vital statistics data from Mexico (1998–2024), we found that deaths from self-inflicted and externally perpetrated violence during pregnancy and up to 1 year post partum have risen markedly, now exceeding some other obstetric causes of death. Incorporating these deaths into the MMR would substantially alter surveillance metrics, revealing mortality currently rendered invisible. Under ICD-MM, suicides during pregnancy or within 42 days post partum should already be counted as maternal deaths;^2^ their exclusion suggests that Mexico’s true MMR is underestimated. For external violence, our findings highlight the need for a global indicator of homicide in pregnancy and post partum to strengthen monitoring and prevention.

These definitional boundaries shape our understanding of the burden of violence-related mortality during pregnancy and the postpartum period. Although self-inflicted and externally perpetrated violence fall on opposite sides of the ICD definition, they often share similar social and structural determinants. In Mexico, spatial correlations suggest overlapping risks: psychological abuse may worsen (perinatal) mental disorders and suicidality,^9^ coercive control and economic abuse may make it more difficult for a woman to seek pregnancy-related care, and pervasive community violence can normalise violence against women,^15^ further constraining women’s ability to leave abusive relationships or households.^14^ The co-occurrence and mutual reinforcement between these forms of violence mean that they cannot be understood in isolation. Modalities of violence provide additional insight into these determinants—most maternal external violence involved firearms, while suicides were most frequently by hanging—patterns consistent across pregnancy and postpartum periods and similar to modalities among all women of reproductive age. This suggests maternal violent deaths reflect broader trends in firearm availability due to conflict in Mexico and their use in the perpetration of gendered violence.^33 34^

Mexico is a distinctive case study because of escalating violence, a strong CRVS system and public discourse on gender-based violence. Yet, the substantial contribution of violence to deaths during pregnancy and post partum is not unique to Mexico. Violence-related maternal deaths are a global blind spot:^35^ homicide was the leading cause of deaths during pregnancy in the USA in 2018/19, exceeding all leading causes of maternal mortality by twofold.^36 37^ Suicide is the leading cause of late maternal death from 6 weeks to 1 year in the UK.^3 38^ Violence related deaths are of similar magnitude to hypertension in Mozambique.^17^ Globally, the contribution of suicide mortality to pregnancy-related and maternal mortality is variable, and data limitations likely result in underestimation.^5 39^

Deaths from suicide and homicide are preventable, and pregnancy and the postpartum period are critical opportunities to identify women at risk. Routine antenatal and postnatal care provide platforms for screening for intimate partner violence, psychosocial distress and suicide risk.^40^ However, while screening with referral may provide avenues for intervention before lethal self-inflicted or external violence occurs, it does little to prevent the problem. Dominant narratives around suicide often psychiatrise the problem, framing it almost invariably as a consequence of mental disorder. This framing obscures the social conditions underlying psychological suffering, such as poverty, social exclusion, childhood sexual abuse and gender-based violence,^17^ and restricted reproductive autonomy, including denial of abortion, particularly among pregnant adolescents.^41^ Effective prevention strategies must therefore extend beyond mental healthcare to tackle these social and structural determinants directly and target the most marginalised groups.^10^

Our comparison of self-inflicted and externally perpetrated violence raises conceptual questions about the operational meaning of ‘aggravation’ within ICD definitions. The ICD defines an aggravated death as one in which pregnancy or its management worsened a pre-existing condition, contributing to the fatal outcome.^2^ Mental disorders can be biologically and socially aggravated by pregnancy, but external violence may also meet this criterion. Pregnancy can increase physiological vulnerability (eg, placental abruption from abdominal trauma).^9^ Beyond biology, pregnancy may also heighten women’s exposure to coercion or control. These forms of ‘social aggravation’ fall outside current ICD interpretation yet may be equally relevant to understanding how pregnancy interacts with violence. Expanding the operationalisation of aggravation could help maternal mortality metrics capture pregnancy-related vulnerability more accurately.

Excluding externally perpetrated deaths from the definition of maternal mortality has far-reaching implications for global goal setting. Because such deaths are omitted from the maternal categories ICD-MM framework, they are also excluded from tracking progress toward SDG 3.1.^1^ Violence against women and girls (VAWG) is absent from the Ending Preventable Maternal Mortality coverage targets,^42^ while SDG 5.2—to eliminate all forms of VAWG—lacks an indicator to measure female homicides or femi(ni)cides. This disjuncture reflects broader institutional siloes: maternal mortality surveillance is led by WHO, and femi(ni)cide monitoring by the UN Office on Drugs and Crime and UN Women.^13^ As a result, violent deaths of pregnant and postpartum women fall through both the maternal health and VAWG agendas, counted in neither. In a post-SDG era, aligning maternal health, violence prevention and mental health strategies could address this blind spot and generate co-benefits for SDG 3.1 and 5.2, as well as SDG 3.4, to reduce premature mortality from non-communicable diseases.

Our findings should be interpreted as a lower bound on the true burden of violence-related maternal mortality in Mexico. Under-reporting affects maternal, suicide and homicide deaths, and misclassification may arise from inconsistent use of the pregnancy checkbox on death certificates,^7^ including potential disincentives to record pregnancy status in cases of external violence or suicide.^5^ Homicide is particularly vulnerable to undercounting in Mexico, given the large number of disappearances, including of women and girls,^43^ some of which may represent unregistered homicides. Some homicides may also be misclassified as suicides. To address challenges in identifying pregnancy status at the time of death, Mexico implemented the *Búsqueda Intencionada y Reclasificación de Muertes Maternas* (BIRMM) methodology in 2003, which was later adopted by the Pan American Health Organization in 2012.^23^ By triangulating clinical histories, autopsy reports and birth or foetal death certificates, BIRMM has improved the detection and classification of maternal deaths, increasing identified cases by 13% between 2006 and 2013.^44^ Nevertheless, although BIRMM strengthens the completeness of maternal death reporting, violence-related maternal deaths are still likely to be underestimated, especially when pregnancy status is unidentified or deaths are unregistered.

### Strengths and limitations

We introduce a novel approach to examine how definitional choices shape international surveillance of deaths during pregnancy and post partum. However, there are several limitations to this study. First, given the robustness of Mexico’s CRVS data and active discourse on gender-based violence, Mexico may represent a best-case scenario for reporting; limitations observed here are likely magnified in less resourced settings with weaker CRVS systems. Second, the absence of medical history prevents the identification of pre-existing mental health conditions. Finally, completeness and accuracy of the pregnancy checkbox may vary over time, potentially influencing trends in pregnancy-related deaths.

## Supplementary material

10.1136/bmjph-2025-004871 online supplemental file 1

## Data Availability

Data are available in a public, open access repository.
