# Supplementary material for: Hidden toll of violent deaths during pregnancy and the postpartum period: a nationwide analysis of Mexican death records
Source: BMJ Public Health. 2026 May 13;4(2):e004871. doi: 10.1136/bmjph-2025-004871 (PMC13182375; doi:10.1136/bmjph-2025-004871)
Supplement: online supplemental file 1 [file bmjph-4-2-s001.pdf]

Appendix

Hidden toll of violent deaths during pregnancy and the postpartum period: a nationwide analysis of Mexican death records

Ursula Gazeley[1,2*], Maria Gargiulo[3*], Hallie Eilerts-Spinelli[4], Anushé Hassan[3], Itzel Díaz Juárez[5], and Alexis Palfreyman[6]

1. Leverhulme Centre for Demographic Science, Nuffield Department of Population Health, University of Oxford, Oxford, United Kingdom

2. Nuffield College, University of Oxford, Oxford, United Kingdom
3. London School of Hygiene and Tropical Medicine, London, United Kingdom
4. Johns Hopkins University, Baltimore, Maryland, United States of America
5. El Colegio de México, Mexico City, Mexico
6. Centre for Impact on Violence and Health, Colombo, Sri Lanka

* These authors contributed equally to this manuscript.

Correspondence: Ursula Gazeley, ursula.gazeley@ndph.ox.ac.uk, Leverhulme Centre for Demographic Science, 42-43 Park End Street, Oxford, OX1 1JD

Table S1. Cause of death groups for analysis

**Category**	**ICD-10 code**
**ICD-MM groups**	
1. Abortive causes	O00–O07
2. Hypertensive disorders of pregnancy	O11–O16
3. Obstetric haemorrhage	O20, O43.2, O44.1, O45, O46, O67, O71.0–O71.4, O71.7, O72
4. Pregnancy-related infection	O23, O85, O86, O91, A34
5. Other direct obstetric	O21.1, O21.2, O22.3, O22.5, O22.8, O22.9, O24.4, O26.6, O26.9, O44.0, O64–O66*, O71.2, O71.5, O71.6, O71.8, O71.9, O73, O75.4, O75.8, O75.9, O87.3, O87.9, O88, O90, F53.0, F53.1
6. Complications of management	O29.0–O29.3, O29.5, O29.6, O29.8, O29.9, O74.0–O74.4, O74.6–O74.9, O89.0–O89.3, O89.5, O89.6, O89.8, O89.9
7. Non-obstetric	O10, O24.0–O24.3, O24.9, O98, O99, C58, D39.2
8. Unknown/undetermined	O95
**Contributory conditions**	
	O08, O21.0, O21.8, O21.9, O22.0–O22.2, O22.4, O25, O26, O28, O29.4, O30–O36, O40, O41.0, O41.8, O41.9, O42, O43.1, O43.8, O43.9, O47, O48, O60–O63, O68–O70, O75.0–O75.2, O75.5–O75.7, O80–O84, O87.0, O87.2, O87.8, O89.4, O92, O94
**Late maternal**	
Late	O96
Sequelae***	O97
**Violence**	
Self-inflicted (and sequelae)	X60–X84, Y87.0

External (and sequelae)	Assault:<br>X85–Y09, Y87.1<br><br>Undetermined intent:**<br>Y10–Y34, Y87.2<br><br>Legal intervention and operations of war:<br>Y35, Y36, Y89.0, Y89.1

*Note: While codes O64–O66 are considered contributory causes, they are included in Group 5 - Other direct obstetric causes of death in line with guidance in the ICD-MM coding manual (p. 58).
**Note: Some deaths coded as undetermined intent may refer to deaths due to self-inflicted violence or accidents. However, in the context of the ongoing forensic crisis and high levels of impunity in Mexico, we decide to group these deaths under external violence.
*** Note: we included 4 deaths which were coded to sequelae of obstetric causes (ICD O97 codes) despite occurring within 1 year post partum.

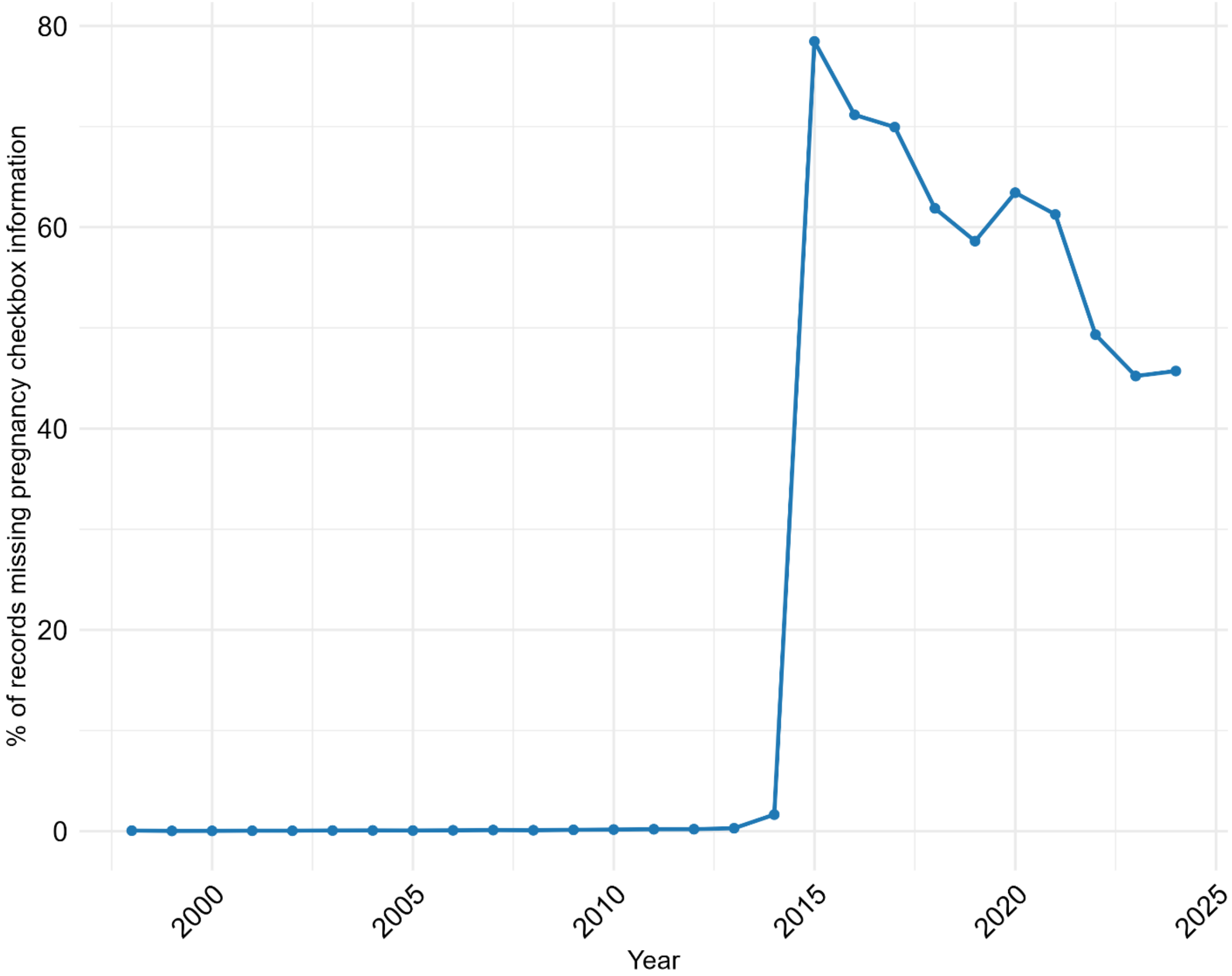

Figure S1. Proportion of death certificates of women aged 10–54 missing pregnancy checkbox information over time (1998–2024)

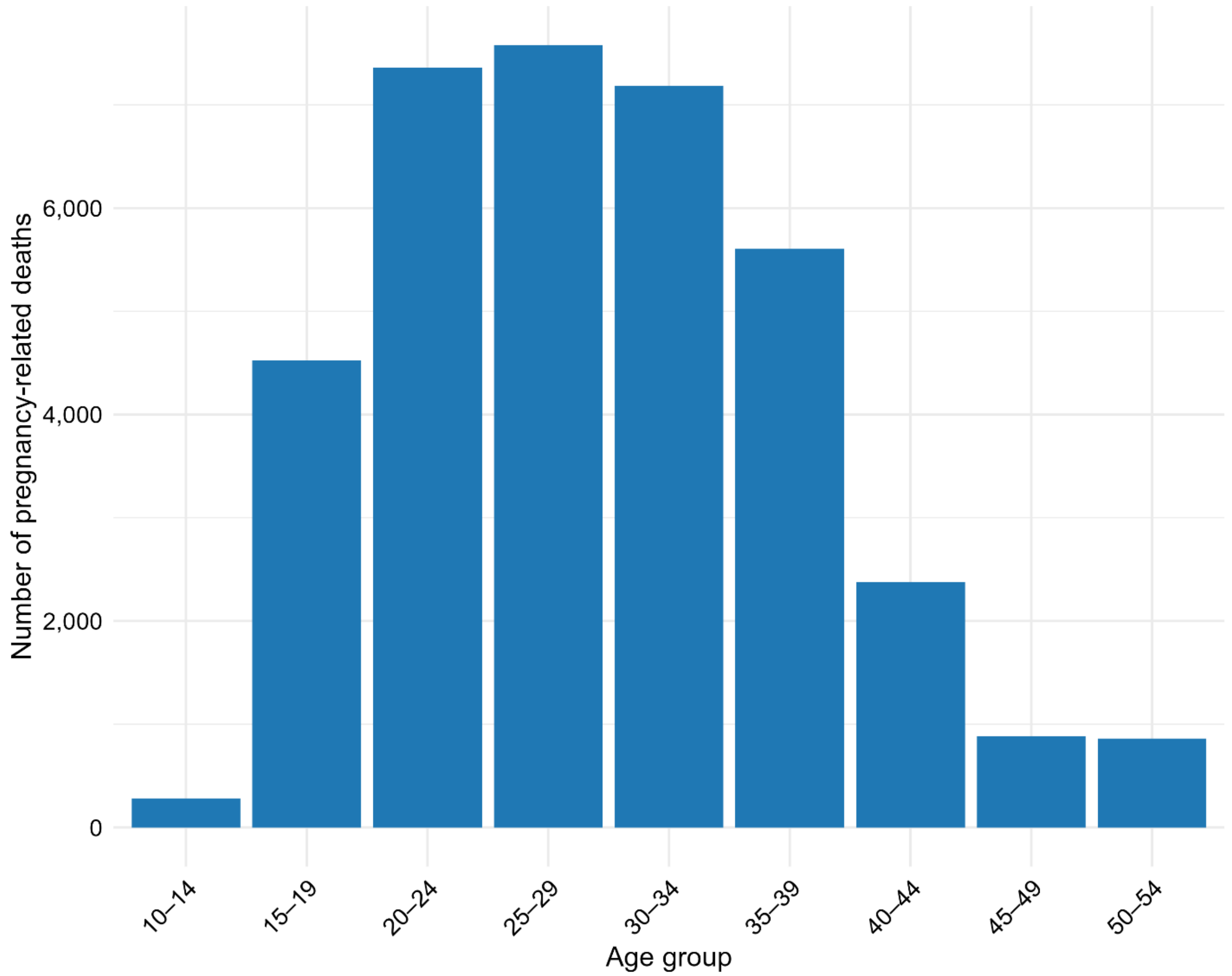

Figure S2. Age distribution of pregnancy-related deaths (1998–2024)

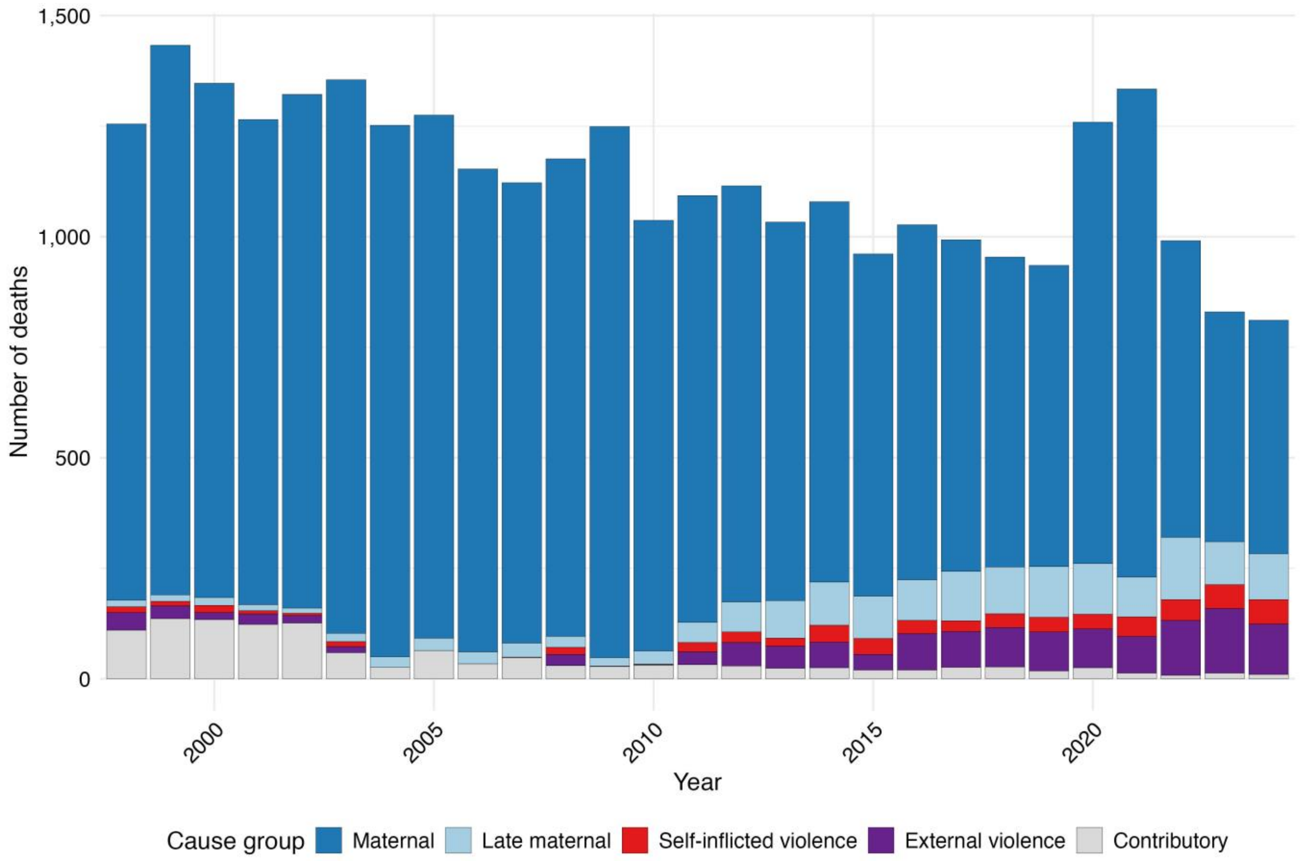

Figure S3. Comparison of deaths due to violence and maternal causes over time (1998–2024)

Note: Late maternal including 4 deaths coded to sequelae of obstetric causes despite occurring within 1 year post partum.

Table S2. Comparison of deaths due to violence and maternal causes over time (1998–2024)

	Maternal	Late maternal[1]	Self-inflicted violence	External violence	Contributory	Year total
**1998**	1,077 (4.2%)	15 (0.9%)	13 (2.3%)	40 (3.1%)	110 (8.9%)	**1,255**
**1999**	1,243 (4.8%)	15 (0.9%)	10 (1.8%)	29 (2.3%)	136 (11.0%)	**1,433**
**2000**	1,163 (4.5%)	18 (1.1%)	16 (2.8%)	16 (1.2%)	134 (10.8%)	**1,347**
**2001**	1,097 (4.2%)	14 (0.9%)	7 (1.2%)	24 (1.9%)	123 (9.9%)	**1,265**
**2002**	1,162 (4.5%)	12 (0.7%)	5 (0.9%)	17 (1.3%)	126 (10.2%)	**1,322**
**2003**	1,252 (4.8%)	19 (1.2%)	11 (1.9%)	14 (1.1%)	59 (4.8%)	**1,355**
**2004**	1,202 (4.6%)	24 (1.5%)	0 (0.0%)	0 (0.0%)	26 (2.1%)	**1,252**
**2005**	1,183 (4.6%)	28 (1.7%)	0 (0.0%)	0 (0.0%)	64 (5.2%)	**1,275**
**2006**	1,092 (4.2%)	27 (1.6%)	0 (0.0%)	0 (0.0%)	34 (2.7%)	**1,153**
**2007**	1,041 (4.0%)	32 (1.9%)	1 (0.2%)	0 (0.0%)	48 (3.9%)	**1,122**
**2008**	1,080 (4.2%)	25 (1.5%)	16 (2.8%)	25 (1.9%)	30 (2.4%)	**1,176**
**2009**	1,201 (4.6%)	19 (1.2%)	0 (0.0%)	1 (0.1%)	28 (2.3%)	**1,249**
**2010**	974 (3.8%)	30 (1.8%)	1 (0.2%)	1 (0.1%)	31 (2.5%)	**1,037**
**2011**	965 (3.7%)	46 (2.8%)	21 (3.7%)	29 (2.3%)	32 (2.6%)	**1,093**
**2012**	941 (3.6%)	68 (4.1%)	24 (4.2%)	53 (4.1%)	29 (2.3%)	**1,115**
**2013**	856 (3.3%)	85 (5.2%)	18 (3.2%)	50 (3.9%)	24 (1.9%)	**1,033**
**2014**	860 (3.3%)	98 (6.0%)	38 (6.7%)	58 (4.5%)	25 (2.0%)	**1,079**
**2015**	774 (3.0%)	96 (5.8%)	36 (6.3%)	35 (2.7%)	20 (1.6%)	**961**
**2016**	803 (3.1%)	92 (5.6%)	30 (5.3%)	82 (6.4%)	20 (1.6%)	**1,027**
**2017**	750 (2.9%)	112 (6.8%)	24 (4.2%)	81 (6.3%)	26 (2.1%)	**993**
**2018**	701 (2.7%)	106 (6.5%)	31 (5.5%)	89 (6.9%)	27 (2.2%)	**954**
**2019**	681 (2.6%)	115 (7.0%)	33 (5.8%)	88 (6.8%)	18 (1.5%)	**935**
**2020**	998 (3.9%)	115 (7.0%)	33 (5.8%)	88 (6.8%)	25 (2.0%)	**1,259**
**2021**	1,104 (4.3%)	90 (5.5%)	44 (7.7%)	83 (6.4%)	13 (1.0%)	**1,334**
**2022**	671 (2.6%)	141 (8.6%)	47 (8.3%)	124 (9.6%)	8 (0.6%)	**991**
**2023**	520 (2.0%)	97 (5.9%)	54 (9.5%)	146 (11.3%)	13 (1.0%)	**830**
**2024**	528 (2.0%)	104 (6.3%)	55 (9.7%)	114 (8.9%)	10 (0.8%)	**811**
**Total**	**25,919**	**1,643**	**568**	**1,287**	**1,239**	**30,656**

[1] Late maternal including 4 deaths coded to sequelae of obstetric causes despite occurring within 1 year post partum.

Table S3. Comparison of self- and externally-inflicted violence by age during pregnancy and postpartum to non-pregnant and postpartum women of reproductive age (10–54 years) (1998–2024)

Age group	Externally inflicted		Self-inflicted	
	Not pregnant or <1 year postpartum	Pregnant or <1 year postpartum	Not pregnant or <1 year postpartum	Pregnant or <1 year postpartum
**10–14**	1,697 (5.6%)	18 (6.4%)	1,657 (5.5%)	19 (6.7%)
**15–19**	5,651 (10.5%)	273 (6%)	4,479 (8.3%)	181 (4%)
**20–24**	7,037 (11.2%)	340 (4.6%)	3,227 (5.1%)	145 (2%)
**25–29**	6,570 (9.4%)	284 (3.7%)	2,468 (3.5%)	106 (1.4%)
**30–34**	5,917 (7.2%)	175 (2.4%)	1,972 (2.4%)	60 (0.8%)
**35–39**	5,130 (5%)	102 (1.8%)	1,592 (1.6%)	31 (0.6%)
**40–44**	4,096 (3.1%)	57 (2.4%)	1,317 (1%)	17 (0.7%)
**45–49**	3,125 (1.7%)	15 (1.7%)	1,108 (0.6%)	5 (0.6%)
**50–54**	2,368 (1%)	23 (2.7%)	825 (0.4%)	4 (0.5%)

Table S4. Deaths by International Classification of Diseases-Maternal Mortality group, Contributory Conditions, Late Maternal and Sequelae, and Violence (1998–2024)

Year	G1: Abortive outcome	G2: Hypertensive diseases	G3: Obstetric haemorrhage	G4: Pregnancy-related infection	G5: Other direct obstetric causes	G6: Complications of management	G7: Non-obstetric causes	G8: Undetermined	Contributory conditions	Late maternal[1]	Violence
1998	85 (6.8%)	383 (30.5%)	244 (19.4%)	56 (4.5%)	158 (12.6%)	2 (0.2%)	145 (11.6%)	4 (0.3%)	110 (8.8%)	15 (1.2%)	53 (4.2%)
1999	94 (6.6%)	461 (32.2%)	291 (20.3%)	55 (3.8%)	157 (11%)	2 (0.1%)	179 (12.5%)	4 (0.3%)	136 (9.5%)	15 (1%)	39 (2.7%)
2000	83 (6.2%)	462 (34.3%)	267 (19.8%)	54 (4%)	142 (10.5%)	2 (0.1%)	151 (11.2%)	2 (0.1%)	134 (9.9%)	18 (1.3%)	32 (2.4%)
2001	68 (5.4%)	454 (35.9%)	271 (21.4%)	41 (3.2%)	148 (11.7%)	0	114 (9%)	1 (0.1%)	123 (9.7%)	14 (1.1%)	31 (2.5%)
2002	95 (7.2%)	416 (31.5%)	248 (18.8%)	57 (4.3%)	131 (9.9%)	2 (0.2%)	210 (15.9%)	3 (0.2%)	126 (9.5%)	12 (0.9%)	22 (1.7%)
2003	87 (6.4%)	409 (30.2%)	366 (27%)	53 (3.9%)	116 (8.6%)	8 (0.6%)	210 (15.5%)	3 (0.2%)	59 (4.4%)	19 (1.4%)	25 (1.8%)
2004	88 (7%)	355 (28.4%)	342 (27.3%)	56 (4.5%)	136 (10.9%)	7 (0.6%)	213 (17%)	5 (0.4%)	26 (2.1%)	24 (1.9%)	0
2005	93 (7.3%)	319 (25%)	328 (25.7%)	45 (3.5%)	141 (11.1%)	3 (0.2%)	250 (19.6%)	4 (0.3%)	64 (5%)	28 (2.2%)	0
2006	94 (8.2%)	332 (28.8%)	275 (23.9%)	31 (2.7%)	117 (10.1%)	8 (0.7%)	229 (19.9%)	6 (0.5%)	34 (2.9%)	27 (2.3%)	0
2007	81 (7.2%)	275 (24.5%)	276 (24.6%)	41 (3.7%)	112 (10%)	7 (0.6%)	249 (22.2%)	0	48 (4.3%)	32 (2.9%)	1 (0.1%)
2008	77 (6.5%)	275 (23.4%)	305 (25.9%)	31 (2.6%)	127 (10.8%)	16 (1.4%)	245 (20.8%)	4 (0.3%)	30 (2.6%)	25 (2.1%)	41 (3.5%)
2009	74 (5.9%)	254 (20.3%)	262 (21%)	40 (3.2%)	104 (8.3%)	9 (0.7%)	449 (35.9%)	9 (0.7%)	28 (2.2%)	19 (1.5%)	1 (0.1%)
2010	93 (9%)	240 (23.1%)	223 (21.5%)	31 (3%)	82 (7.9%)	11 (1.1%)	290 (28%)	4 (0.4%)	31 (3%)	30 (2.9%)	2 (0.2%)
2011	75 (6.9%)	230 (21%)	239 (21.9%)	40 (3.7%)	87 (8%)	9 (0.8%)	283 (25.9%)	2 (0.2%)	32 (2.9%)	46 (4.2%)	50 (4.6%)
2012	79 (7.1%)	238 (21.3%)	213 (19.1%)	30 (2.7%)	97 (8.7%)	9 (0.8%)	275 (24.7%)	0	29 (2.6%)	68 (6.1%)	77 (6.9%)
2013	77 (7.5%)	202 (19.6%)	185 (17.9%)	25 (2.4%)	90 (8.7%)	4 (0.4%)	270 (26.1%)	3 (0.3%)	24 (2.3%)	85 (8.2%)	68 (6.6%)
2014	81 (7.5%)	176 (16.3%)	162 (15%)	26 (2.4%)	102 (9.5%)	4 (0.4%)	306 (28.4%)	3 (0.3%)	25 (2.3%)	98 (9.1%)	96 (8.9%)
2015	72 (7.5%)	162 (16.9%)	145 (15.1%)	20 (2.1%)	112 (11.7%)	5 (0.5%)	256 (26.6%)	2 (0.2%)	20 (2.1%)	96 (10%)	71 (7.4%)
2016	70 (6.8%)	195 (19%)	162 (15.8%)	23 (2.2%)	86 (8.4%)	4 (0.4%)	256 (24.9%)	7 (0.7%)	20 (1.9%)	92 (9%)	112 (10.9%)
2017	75 (7.6%)	162 (16.3%)	155 (15.6%)	37 (3.7%)	81 (8.2%)	1 (0.1%)	234 (23.6%)	5 (0.5%)	26 (2.6%)	112 (11.3%)	105 (10.6%)
2018	67 (7%)	155 (16.2%)	138 (14.5%)	21 (2.2%)	89 (9.3%)	4 (0.4%)	221 (23.2%)	6 (0.6%)	27 (2.8%)	106 (11.1%)	120 (12.6%)

2019	70 (7.5%)	156 (16.7%)	125 (13.4%)	21 (2.2%)	85 (9.1%)	3 (0.3%)	218 (23.3%)	3 (0.3%)	18 (1.9%)	115 (12.3%)	121 (12.9%)
2020	86 (6.8%)	167 (13.3%)	176 (14%)	24 (1.9%)	75 (6%)	4 (0.3%)	460 (36.5%)	6 (0.5%)	25 (2%)	115 (9.1%)	121 (9.6%)
2021	54 (4%)	154 (11.5%)	126 (9.4%)	17 (1.3%)	83 (6.2%)	5 (0.4%)	659 (49.4%)	6 (0.4%)	13 (1%)	90 (6.7%)	127 (9.5%)
2022	70 (7.1%)	135 (13.6%)	125 (12.6%)	17 (1.7%)	84 (8.5%)	3 (0.3%)	234 (23.6%)	3 (0.3%)	8 (0.8%)	141 (14.2%)	171 (17.3%)
2023	53 (6.4%)	109 (13.1%)	110 (13.3%)	19 (2.3%)	67 (8.1%)	6 (0.7%)	155 (18.7%)	1 (0.1%)	13 (1.6%)	97 (11.7%)	200 (24.1%)
2024	60 (7.4%)	100 (12.3%)	104 (12.8%)	18 (2.2%)	58 (7.2%)	4 (0.5%)	179 (22.1%)	5 (0.6%)	10 (1.2%)	104 (12.8%)	169 (20.8%)
**Total**	**2,101**	**6,976**	**5,863**	**929**	**2,867**	**142**	**6,940**	**101**	**1,239**	**1,643**	**1,855**

[1] Late maternal including 4 deaths coded to sequelae of obstetric causes despite occurring within 1 year post partum.

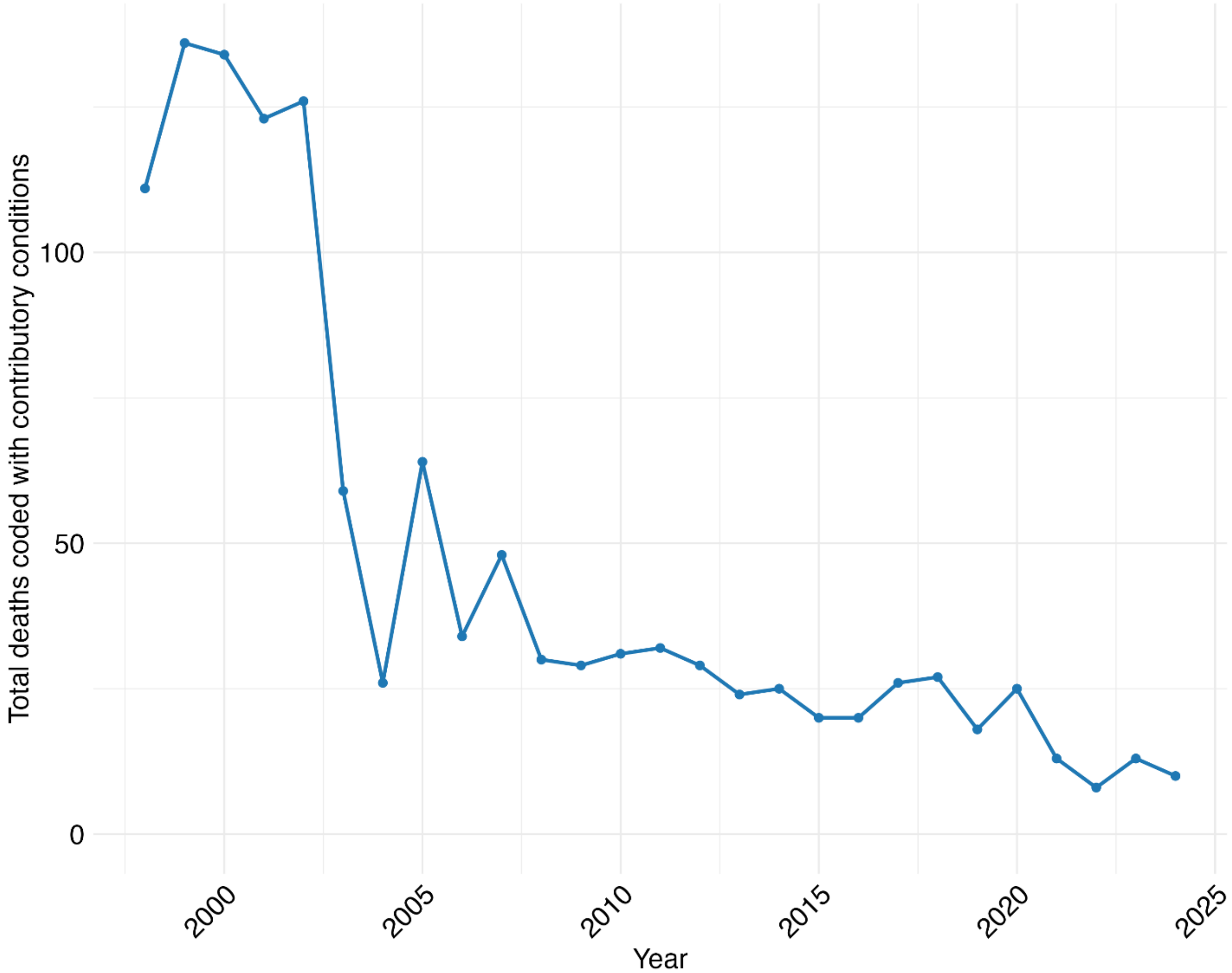

Figure S4. Deaths of pregnant and postpartum women coded to contributory conditions over time (1998–2024)

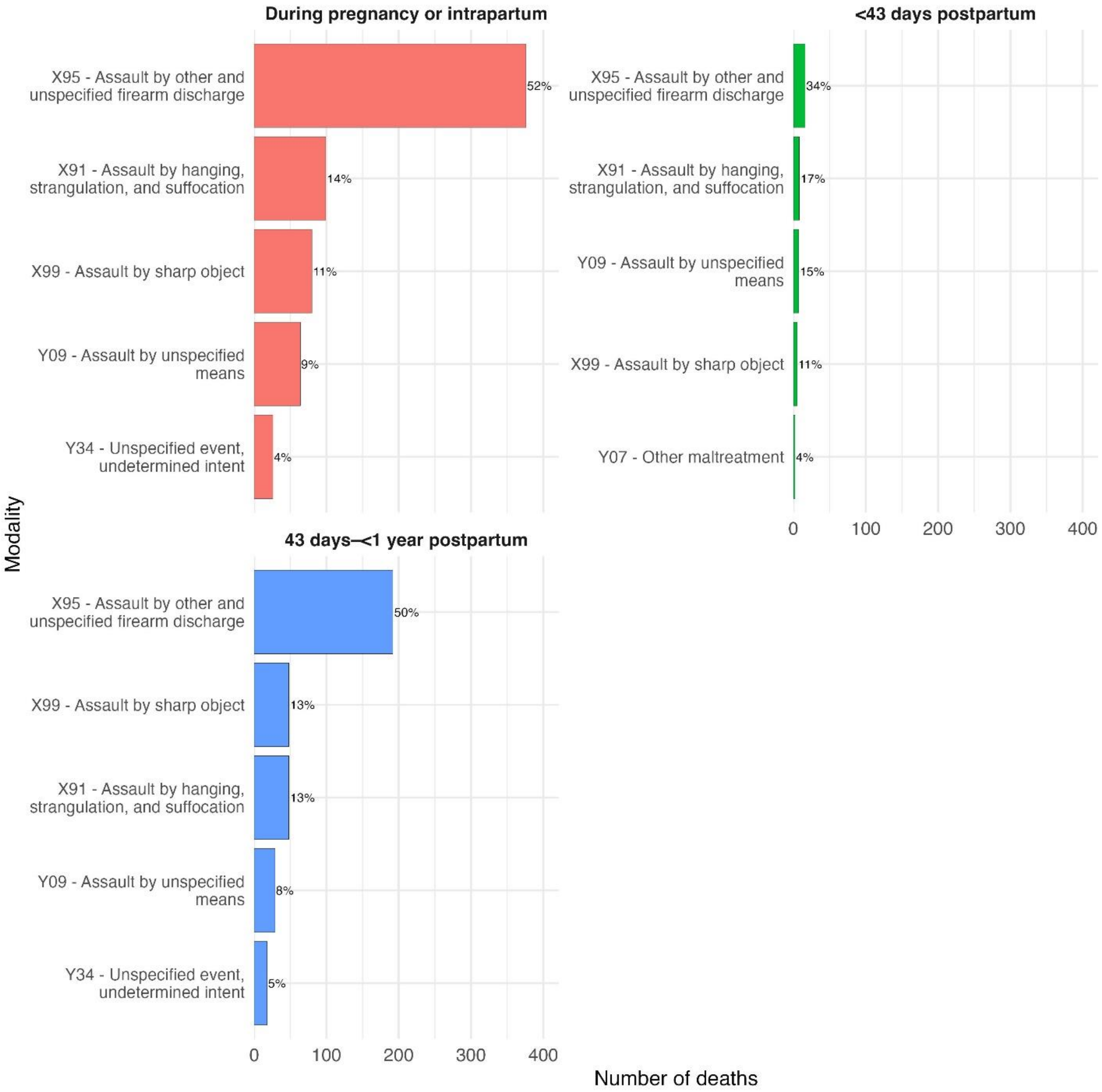

Figure S5. Modalities of external violence with timing disaggregated (2004–2024)

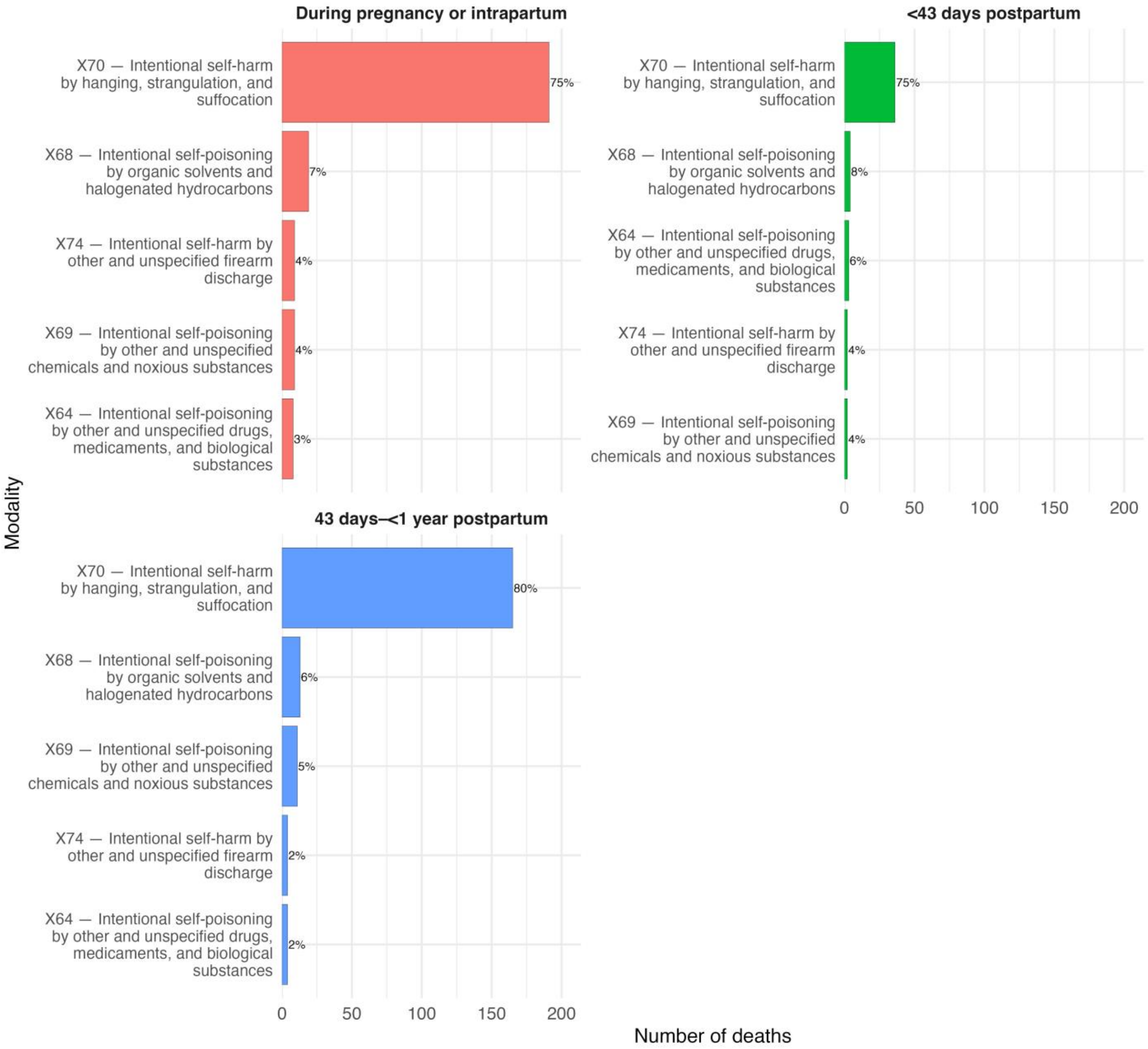

Figure S6. Modalities of self-inflicted violence with timing disaggregated (2004–2024)

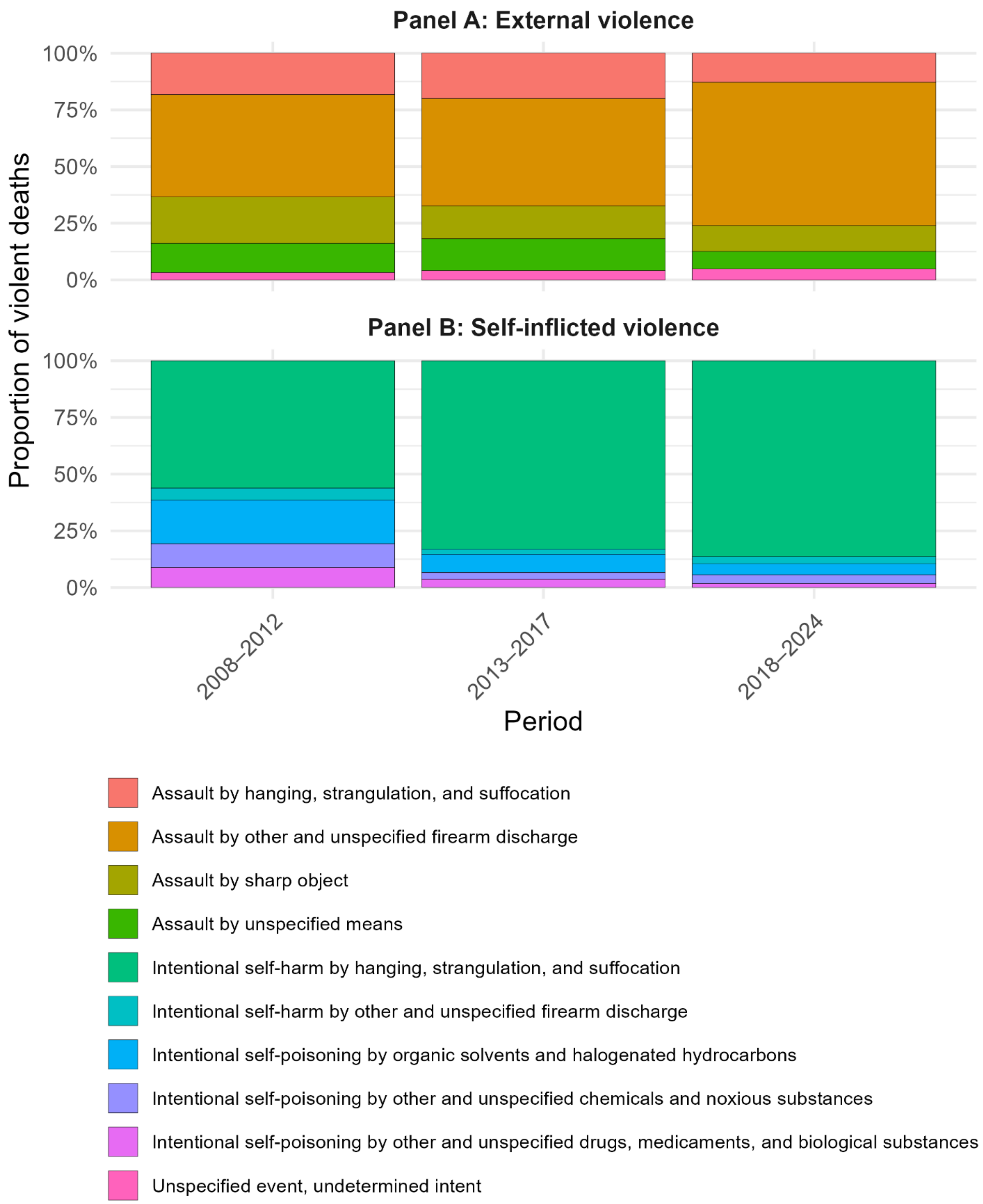

Figure S7. Changing modalities of violent deaths over time (2008–2024)

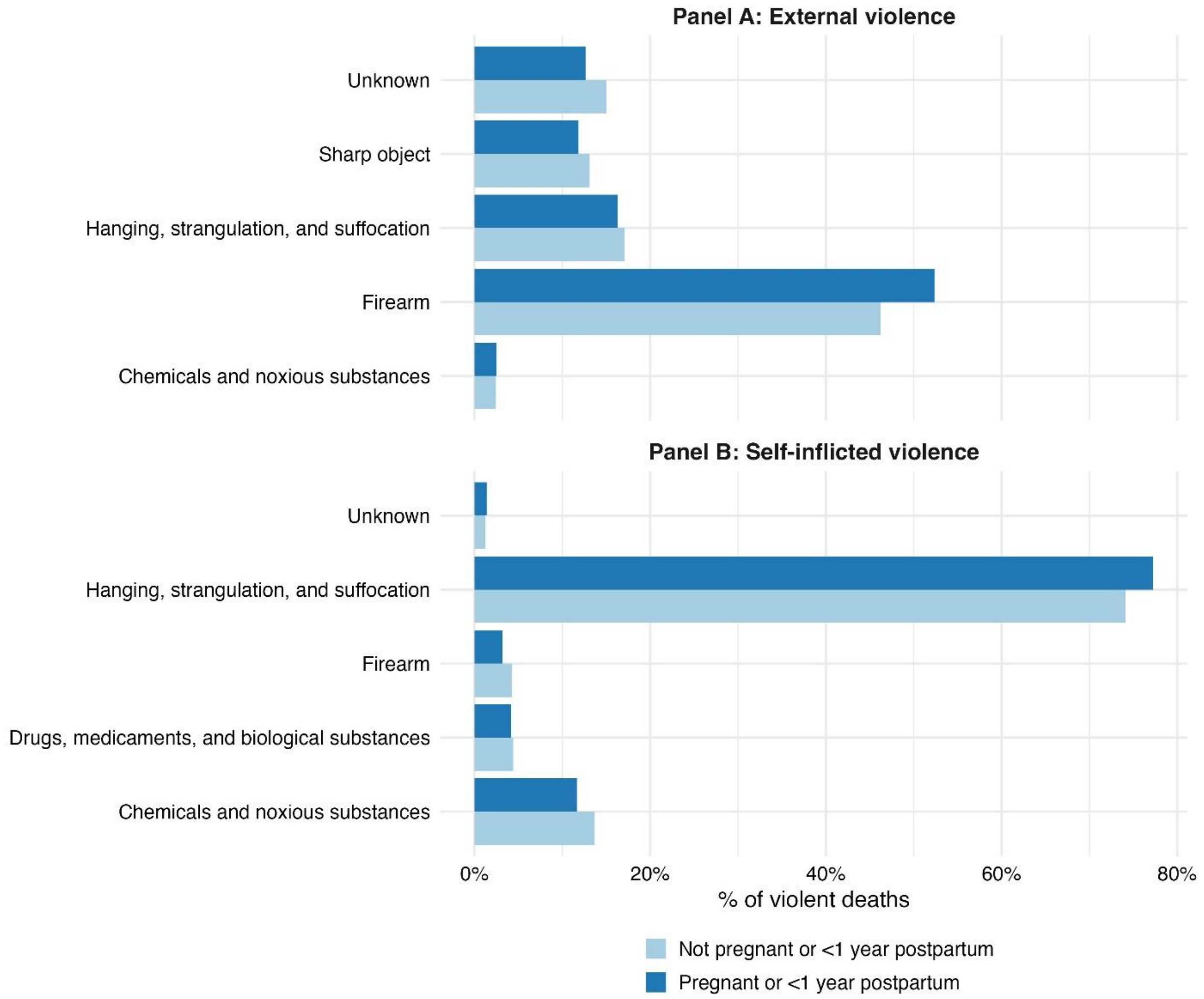

Figure S8. Modalities of violence by pregnancy status versus women of reproductive age (1998–2024)

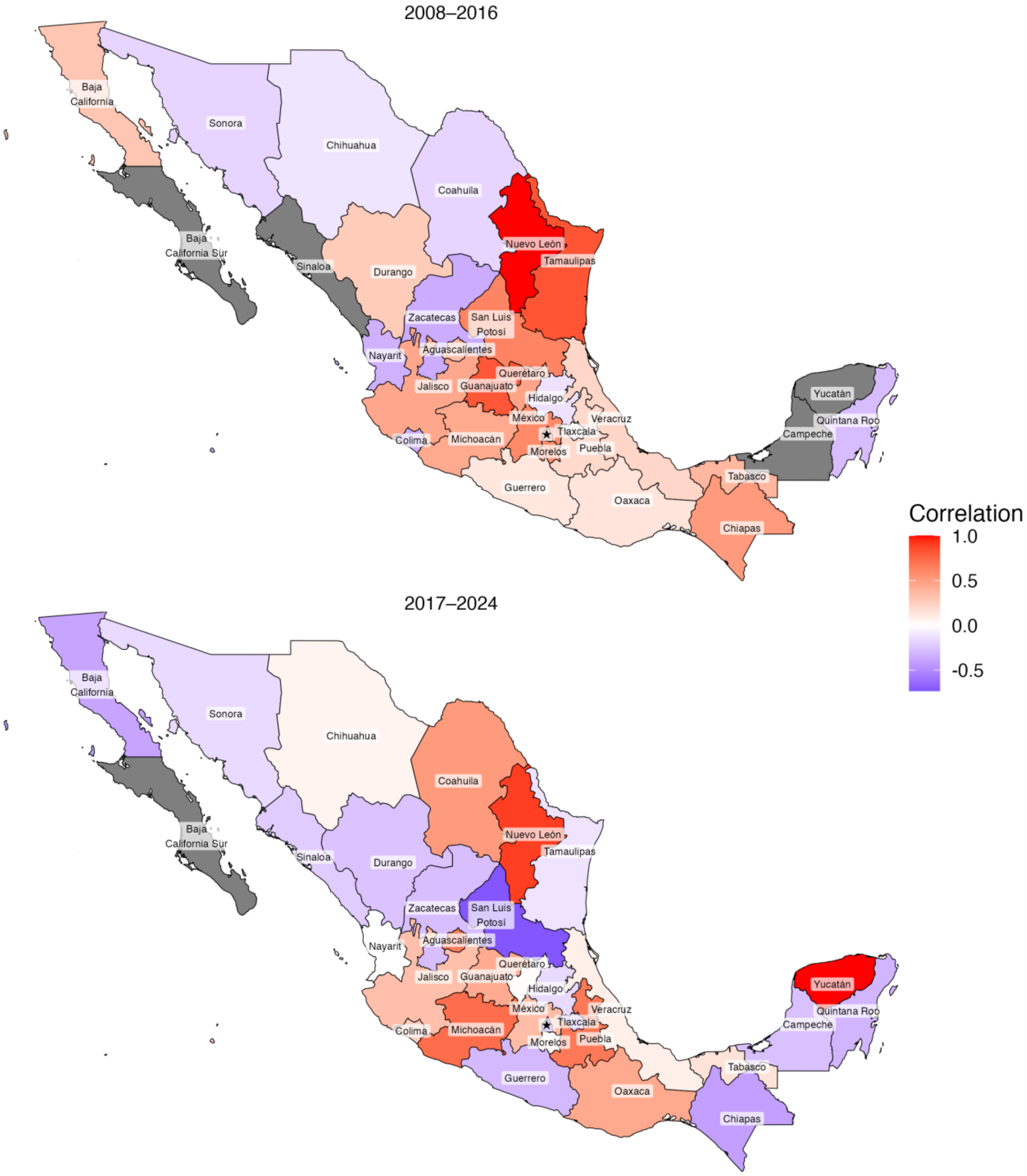

Figure S9. Spatial correlation between self-inflicted and externally perpetrated violence by period (2008–2024)

Note: the star represents Mexico City.

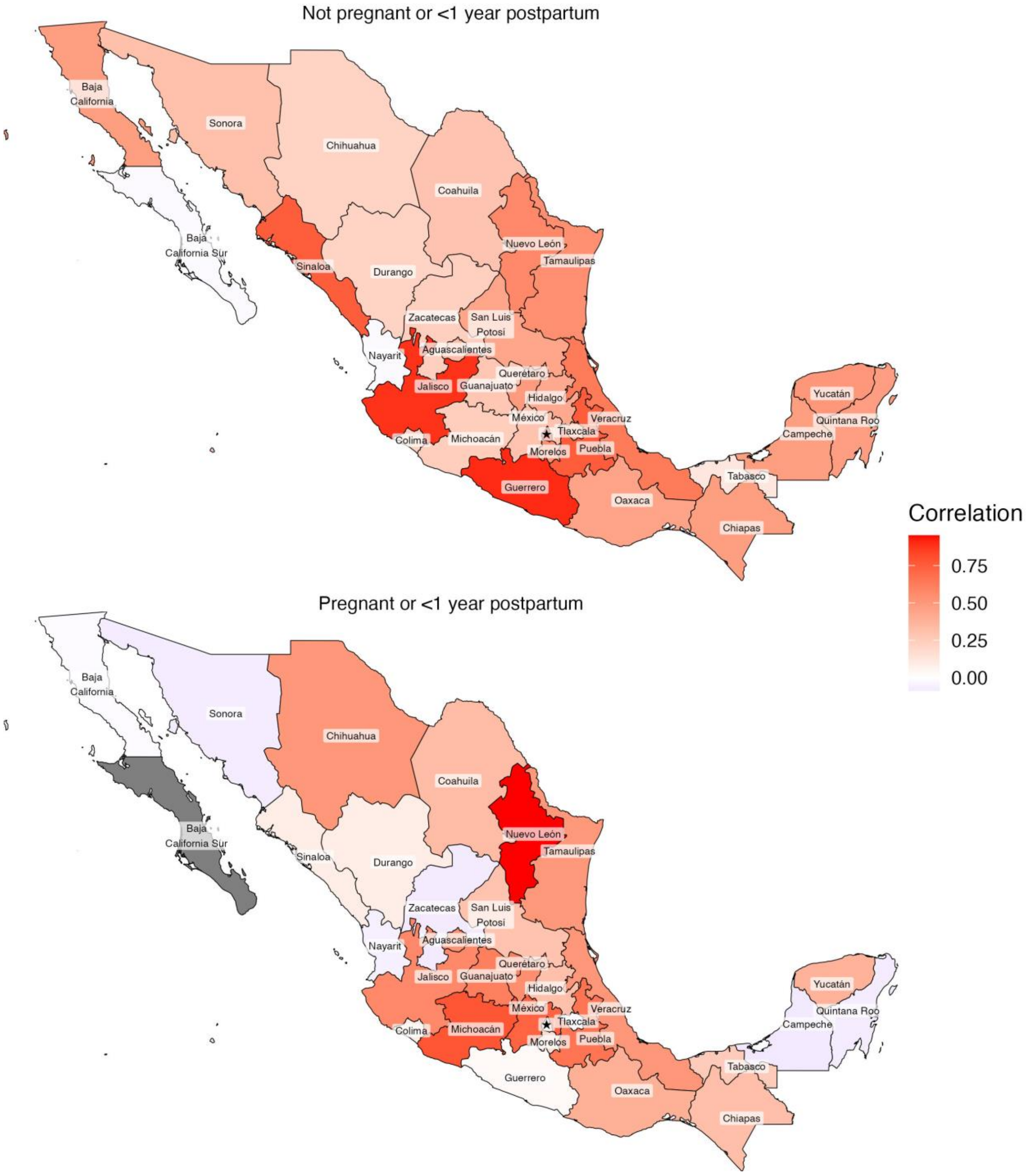

Figure S10. Spatial correlation between self-inflicted and externally perpetrated violence by pregnancy status (1998–2024)

Note: the star represents Mexico City.
